# From Cell Walls to Food Products: Health Benefits, Functional Properties and Future Challenges of Yeast β-Glucans

**DOI:** 10.3390/nu18050836

**Published:** 2026-03-04

**Authors:** Kalliopi-Maria Makriyanni, Amalia E. Yanni

**Affiliations:** Laboratory of Chemistry-Biochemistry-Physical Chemistry of Foods, Department of Nutrition and Dietetics, Harokopio University, 17671 Athens, Greece; poppymakr219@gmail.com

**Keywords:** yeast β-glucans, *Saccharomyces cerevisiae*, immunomodulation, probiotic effects, food fortification, assessment methods, clinical studies, in vitro digestion

## Abstract

Yeast β-glucans are bioactive polysaccharides derived primarily from the cell walls of *Saccharomyces cerevisiae*. They are widely recognized for their immunomodulatory, antioxidant, and anti-inflammatory actions as well as for their probiotic effects. Their addition to food products has gained growing interest owing to their ability to promote health as well as to enhance sensorial and technological attributes of foods. The aim of this narrative review is to present the health benefits of yeast β-glucans according to the mechanisms taking place, compare them to other biomolecules with analogous health-promoting effects, and summarize the existing knowledge on their incorporation into various food matrices. Focus is also given to clinical trials using foods enriched with yeast β-glucans as well as in vitro digestion studies of such foods. In addition, research interest extends to the methods of yeast β-glucan assessment in food products. Despite the promising results so far, significant challenges remain, including variability in study design, limited translational evidence from in vitro studies, and the lack of standardized protocols for determination across various food categories. Overall, the reviewed literature supports their growing potential as valuable components in the design of functional foods. Ongoing research and advancement should prioritize well-designed human trials, standardized production protocols and deeper structure–function relationship investigation in order to further reveal their contribution across a wide range of applications, reinforcing both consumer health and innovation within the food industry.

## 1. Introduction

In recent years, there is growing consumer awareness of the link between nutrition and health that has fueled the rapid expansion of the functional foods sector, encompassing products that offer health benefits beyond their basic nutritional value [1]. Globally, the functional foods market was valued at approximately 329.65 billion USD in 2024 and is projected to reach 586.06 billion USD by 2030, reflecting strong demand for health-oriented food products [2].

Among the various bioactive compounds explored for functional food applications, β-glucans have attracted considerable scientific and industrial interest due to their well-documented physiological effects. B-glucans are naturally occurring polysaccharides, consisting of heterogenous D-glucose groups/units. They are found frequently in the cell wall of cereals such as oats, barley and wheat. They are also present in certain microorganisms such as fungi (including mushrooms), bacteria, and algae [3]. While β-glucans are present in various sources, yeast-derived β-glucans, particularly those obtained from *S. cerevisiae*, possess distinct structural characteristics that confer unique biological activities [4].

Yeast β-glucans are non-starch polysaccharides located primarily in their cell walls, consisting mainly of a β-(1→3)-glucan backbone with β-(1→6) side chains [5]. This specific molecular architecture is largely responsible for their immunomodulatory, antioxidant, and anti-inflammatory properties, which have been investigated in both experimental and clinical studies. Numerous studies have demonstrated that yeast β-glucans can interact with immune cell receptors, thereby enhancing innate immune responses and contributing to host defense mechanisms [6].

Beyond their biological effects, yeast β-glucans have gained increasing attention in the food science field due to their technological and sensorial properties. Their incorporation into food matrices can influence texture, viscosity, water-holding capacity, and stability, making them attractive ingredients for product reformulation and nutritional enhancement. Consequently, yeast β-glucans have been incorporated into a variety of foods, including bakery products, beverages, dairy-based formulations, and starch-rich systems, with the dual aim of improving health outcomes and maintaining or enhancing product quality [7].

The article aims to provide a comprehensive overview of the topic. Since the literature concerning applications of yeast β-glucans in foods is extensive but not boundless, a narrative approach was selected as a more adjustable and dynamic way of presenting the existing data. This review compiles recent findings from studies, clinical trials, reviews, and meta-analyses on the incorporation of yeast β-glucans into various foods and their influence on both human health and food physicochemical characteristics. An analysis of the yeast β-glucan assessment methods was also performed.

## 2. Materials and Methods

Records selected for this review were drawn from the following databases: Pubmed, ScienceDirect, Google Scholar and Scopus. Articles published up to October 2025 were reviewed, emphasizing those released since 2015, with the exception of some well-established works. Articles were selected based on relevance to the review topic, and the most recent and impactful studies were prioritized. The key words used in the research included “yeast β-glucans”, “*Saccharomyces cerevisiae*”, “immunomodulation”, “probiotic effects”, “food fortification”, “assessment methods”, “clinical studies”, and “in vitro digestion”. Among the exclusion criteria were studies using yeast β-glucan supplementation in preparation of capsule forms. Additionally, studies inaccessible in full text or not written in English were not considered.

## 3. Physicochemical Properties and Structural Characteristics of Yeast β-Glucans

**Figure 1:**
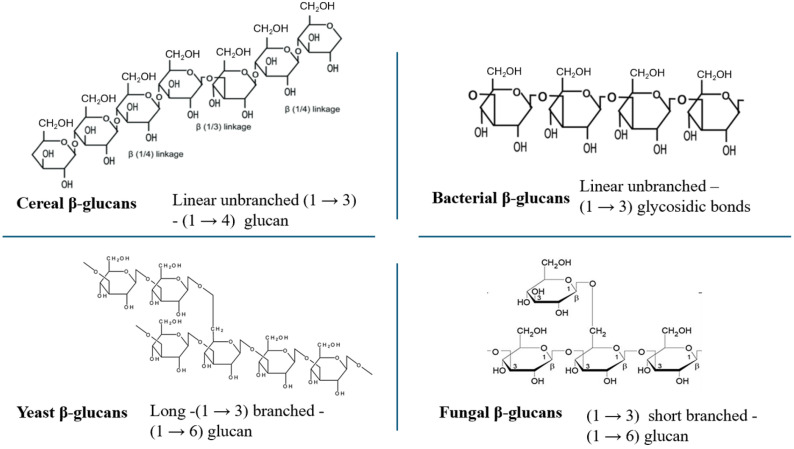
Structures of β-glucans derived from different sources.

In relation to the structure of β-glucans, they are linked mainly by β-(1→3) glycosidic bonds, along with (1→4) or (1→6) glycosidic bonds, forming a single or a triple helix, under the effect of hydrogen bonds [8]. Cereal β-glucans consist of glucose molecules combined with β-(1→3), (1→4) glycosidic linkages. Bacterial β-glucans form a linear β-(1→3) structure, while most yeast and filamentous fungi β-glucans are formed by (1→3), (1→6) linked branches. Filamentous fungi consist of short (1→6) branched (1→3) β-glucans while yeasts consist of long (1→6) branched (1→3) β-glucans [9]. The molecular weight of β-glucans varies relying on the origin. Precisely, β-glucans derived from cereals have higher molecular weight which can reach up to 1600 kDa for oat and 2700 kDa for barley [9]. As for yeasts and filamentous mushrooms, they often show a broader distribution of molecular weights, depending on the species, which may range from a few kDa to hundreds of kDa [9,10]. The molecular weight of bacteria β-glucan with linear structure varies from 5 to thousand kDa whereas most of algae have very low MW (<5 kDa) [5]. The different β-glucan structures are summarized in Figure 1.

B-glucans derived from cereal sources mainly exert metabolic effects, such as cholesterol lowering activity, postprandial glycemic control and gut microbiome modulation while non-cereal-derived β-glucans typically exert their effects by interacting with the immune system [8].

Yeast is a major source of β-glucans, being most prevalent among cell walls of *S. cerevisiae*. *S. cerevisiae* is a species of yeast, commonly known as baker’s yeast or brewer’s yeast which is widely used in wine and beer fermentation processes. Its cell wall is made up of three distinct layers. While the outer layer consists of proteoglycans, the central and the inner stratum contain soluble and insoluble β-glucans, respectively [11]. The cell wall is primarily made up of polysaccharides (around 85%) and proteins (around 15%). β-glucan in *S. cerevisiae* comprises 65–90% of total yeast polysaccharide [12]. As previously mentioned, yeast predominantly includes (1→3)-β-glucan-containing β-(1→6)-branches [4]. In the *S. cerevisiae* cell wall, glucans consist of linear β-(1→3)-linked glucose chains, approximately 30 units long, connected by β-(1→6) branching points. It is estimated that the presence of (1→3) β-glucan in *S. cerevisiae* can reach up to 55% of the cell wall, while the (1→6) β-glucan ranges from 10 to 15% of it [11]. The branched linkage of this type of β-glucans has recognized bioactive properties, such as immunomodulatory effects [8]. Over half of the β-D-glucans in yeasts consist of long chains containing 1500 glucose units connected by β-(1→3) glycosidic bonds, with a molecular weight reaching up to 240 kDa [13]. The distinct structure of yeast β-glucans enables interaction with hydroxyl groups, forming a simple or a triple helix, or irregular constructions [14]. This conformation underlies the high molecular weight and broad molecular weight distribution observed in this type of glucans [13]. B-glucan’s structure, branching degree, and glucose molecular number as well as preparation methods can affect its physicochemical characteristics and functionality [15]. Yeast β-glucans are mostly insoluble in water and almost all solvents. This chemical nature of particulate β-glucans limits their applicability in numerous medical and food-related uses [13]. Due to their insolubility, yeast β-glucans are not able to form viscous solutions. However, this lower viscosity is useful in food industry regarding beverages and liquids in order to enhance stability and prevent phase separation [16]. They are frequently incorporated into food formulations for their ability to enhance stability in liquid and semi-liquid products such as soups, sauces, and beverages [17].

## 4. Biological Activities and Health-Promoting Effects of Yeast β-Glucans

Yeast β-glucans have been studied not only for their physicochemical characteristics but also for their biological activities. These include mainly immunomodulation and antioxidant and anti-inflammatory capacity. Further attributes include antiviral and antibacterial action, wound healing capacity, gut microbiota regulation and possible anti-tumor action [9,18].

Although immune response, oxidative stress, and inflammation are closely interconnected physiological processes and influence each other, they have been studied separately.

**Figure 2:**
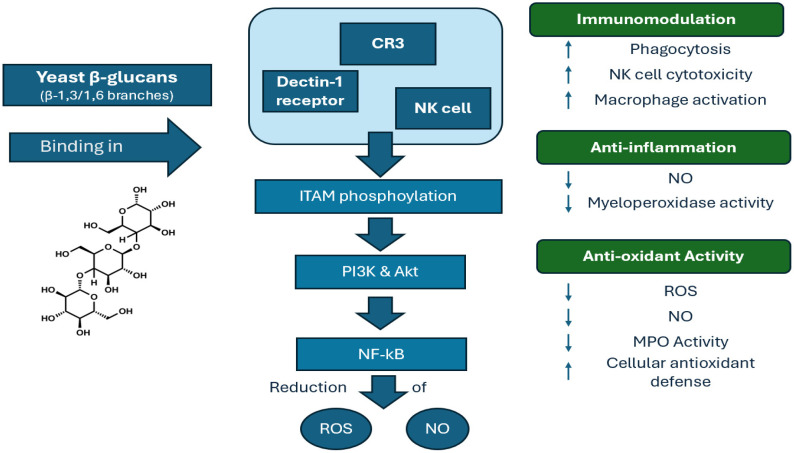
Schematic representation of the immunomodulatory, anti-inflammatory, and antioxidant effects of yeast β-glucans. CR3: complement receptor 3; NO: nitric oxide; MPO: myeloperoxidase; NK cells: natural killer cells; NO: nitric oxide; PI3K: phosphoinositide 3-kinase; Akt: protein kinase B; ROS: reactive oxygen species; ITAM: immunoreceptor tyrosine-based activation motif. (upwards arrow means increase and downwards decrease).

There is evidence for an immunity-boosting capacity of yeast β-glucans. More precisely, they are capable of binding to specific recognition receptors such as Dectin-1 receptors, CR3 and other immune receptors. When binding to Dectin-1, they trigger a signaling cascade inside the immune cell, leading to the phosphorylation of tyrosine-based activation motif (ITAM) and thus to the stimulation of PI3K/Akt pathway, which is crucial for immune cell functions [8]. Thus, they promote the activation of cytokines, B-cells and T-cells and stimulate a series of immune cell responses [4,18]. Research also suggests that soluble yeast β-glucans are highly effective in decreasing free radicals resulting in reducing oxidative stress and protecting cell from damage [14]. Guo et al. investigated the antioxidant capacity of β-glucans and mannans from yeast cell walls by adding them to a porcine jejunal epithelial cell line. The results showed an enhanced cellular glutathione concentration and reduced the ROS content, regulating autophagy-related damage [19]. When analyzing the underlying mechanism in yeast β-glucan from *S. cerevisiae* cells, the nuclear factor erythroid 2-related factor 2 (Nrf2)/heme oxygenase-1 (HO-1) seems to be stimulated. The Dectin-1 signaling mechanism plays a key role by lowering the oxidative damage [20]. Regarding inflammation, several studies, mainly in vitro experiments and animal model studies, have been conducted with positive anti-inflammatory effects derived from yeast β-glucans [21]. The principal findings include reduction in inflammatory enzymes and mediators such as myeloperoxidase activity (MPO) as well as a decrease in nitrιc oxide (NO) and malondialdehyde levels [22]. Particularly, a decrease in myeloperoxidase, eosinophil peroxidase and N-acetyl-b-D-glucosaminosidase levels was observed in mice with acute colitis receiving orally administered yeast β-glucans for seven days [23]. Results from in vitro studies reveal also a possible anti-inflammatory action. Yeast β-glucan was added to RAW 264.7 macrophages after fermentation with gut bacteria. mRNA expression of TLR4/MyD88 seemed to decrease while pro-inflammatory agents induced by lipopolysaccharide LPS were suppressed. Yeast β-glucan anti-inflammatory effects acted through reducing intracellular ROS synthesis and downregulating the mRNA expression of the NF-κB and JNK signaling pathways [24]. When yeast β-glucans were applied to tumor cells for two weeks, there was a decrease in pro-inflammatory cytokines (TNF-α, IL-6) and an increase in anti-inflammatory agents (IL-10, TGF). In addition, they resulted in tumor growth retardation and boosted NK-cell activation and cytotoxicity against tumor cells [25]. A schematic overview of the mechanisms underlying the effects of yeast β-glucans on immune activity is presented in Figure 2.

## 5. Legal Framework

Regulatory approaches to yeast β-glucans vary across regions. In 2011, yeast β-glucans derived from *S. cerevisiae* have been approved by the European Food Safety Authority (EFSA) as novel food ingredients, as they may offer potential health benefits to consumers [26]. In 2017, the European Commission updated the approval under the new regulatory framework (EU 2017/2078), by expanding market access while uploading safety and labeling standards and defining the maximum levels. More precisely, β-glucans from yeast-derived products are approved for use as food supplements at a maximum dose of 1.275 g per day for individuals over 12 years old and adults and 0.675 g per day for children under 12 years old. The document extends the use of β-glucans to other foods such as beverages, cereals, bakery products, etc., highlighting the maximum dose that can be added to them [27]. Yeast β-glucans have also been classified as Generally Recognized As Safe (GRAS) by the U.S. FDA (2008) [28], under correct manufacturing practices. Foods with yeast β-glucan should meet proper food labeling and safety rules. Concerning the World Health Organization, a high amount of fiber intake is suggested daily in the context of healthy nutrition, reaching up to over 25 g of naturally occurring total fiber intake. However, no specific guidelines or requirements are met for β-glucans (including oat, barley, yeasts and other sources) [29]. In Canada, yeast β-glucans are regulated under the framework of novel foods and natural health products (NHPs). In this context, a commercially available preparation named Sourvisiae^®^ has been designed and has been evaluated by Health Canada for safety as a novel food ingredient, indicating that it can be safely consumed within recommended use levels. However, no standardized daily intake or officially approved health claims exist for yeast β-glucans in Canada [30]. In Australia and New Zealand, products containing yeast β-glucans (*S. cerevisiae*) are regulated by the Therapeutic Goods Administration (TGA), and therefore they should meet applicable standards for product registration, labeling, manufacturing, and indications [31].

## 6. Comparative Evaluation of Yeast β-Glucans and Other Functional Food Biomolecules

Yeast β-glucans, primarily obtained from *S. cerevisiae*, have undoubtedly received considerable attention as functional ingredients due to their extensively studied biological properties and mainly immunomodulation. Studies have demonstrated their health-promoting effects including their anti-cancer effects and reduction in fatigue and infections [32]. Clinical investigations have consistently reported a favorable tolerability profile, with minimal adverse effects even at relatively high intake levels [33]. As far as safety is concerned, regulatory assessments, such as EFSA’s evaluation of yeast β-glucans as a novel food ingredient, have concluded that the source, characterization, and production processes do not give reasons for safety concerns at the proposed usage levels, and allergenic risk is not higher than that associated with other yeast products [27]. Both the advantages and the limitations of yeast β-glucans become more evident when evaluated alongside other structurally related and immunologically active biomolecules. Biomolecules frequently employed for health promotion through immunomodulation such as inulin, fructooligosaccharides (FOS), chitosan and polyphenols present alternative mechanisms of action and production frameworks.

### 6.1. Mechanisms of Action

The (1→3), (1→6) branching of yeast β-glucans enables direct interaction with immune cell receptors such as Dectin-1 and CR3, mediating immune responses [4]. Conversely, inulin and FOS are non-digestible oligosaccharides that exert their effects predominantly through selective fermentation by the gut microbiota. Immune homeostasis is indirectly affected through microbial composition and gut-produced SCFAs, pathways that do not typically engage immune cell receptors directly in the manner observed for yeast β-glucans [34]. Chitosan is a linear polysaccharide derived from chitin. Its biological function is strongly dependent on its physicochemical characteristics. The immunomodulatory properties of chitosan have been reviewed, showing that its interaction with immune cells can vary and depend on physicochemical properties [35]. Polyphenols are known for their antioxidant activity by neutralizing reactive oxygen species and regulating pro-inflammatory signals, including NF-κB activation. As a diverse group of plant secondary metabolites, they modulate oxidative and inflammatory responses by acting on redox-sensitive signals and transcriptional regulators. Their physiological effects are strongly shaped by intestinal bioavailability and microbiota-mediated biotransformation [36].

### 6.2. Safety, Sustainability and Cost

Regarding safety perspective, although yeast beta-glucans exhibit a well-tolerated profile at nutritionally relevant doses, their biological activity is influenced by physicochemical characteristics as well as extraction and purification procedures. These parameters can have an impact on reproducibility, complicating standardization across different commercial and experimental preparations [37]. Inulin and FOS are also generally regarded as safe at nutritionally relevant doses. However, dose-dependent tolerance has been observed across studies of inulin consumption [38], and enteral symptoms such as bloating and flatulence have been noticed in clinical studies focusing on FOS consumption [39]. Chitosan is generally well tolerated although there are some allergens concerns in some individuals with shellfish hypersensitivity [40]. Polyphenols derived from food are generally regarded as safe. Nevertheless, high doses from supplements, enhanced bioavailability formulations, or interaction with certain medication can have potential adverse effects that are increasingly recognized in the recent literature [41].

Yeast β-glucans are derived from microbiome fermentation which enables reproducible and high-yield β-glucan production. This offers a sustainable and climate-independent source in contrast with plant- or animal-derived materials [42]. Probiotic materials such as inulin and FOS are produced from plants or enzymatically. These production systems are mature and economically efficient, supported by stable and well-developed supply chains. However, they remain dependent on agricultural frameworks that are inherently influenced by seasonal patterns and climatic conditions [43]. Chitosan extraction is based on marine crustacean shells, and this procedure typically requires energy-demanding and chemically intensive procedures. Furthermore, raw material availability is contingent upon fluctuations in marine harvesting and seafood industry outputs [44]. Polyphenols are mainly sourced from crops. Tea leaves, grapes, and various fruits are intrinsically dependent on agricultural cultivation and post-harvest processing conditions. Their availability, compositional profile, and overall yield are influenced by factors including crop variety, climate, soil conditions, and seasonal variability. Furthermore, the production of concentrated or high-purity polyphenol extracts intended for nutraceutical applications typically requires sophisticated extraction and purification techniques, which can increase manufacturing costs and introduce additional variability in final composition [45]. Returning to yeast β-glucans, although large-scale fermentation systems are well established and readily scalable, downstream processing, particularly cell wall disruption, extraction, and purification, constitute a major component of production expenses. Moreover, differences in processing strategies can lead to variability in β-glucan yield, structural integrity, and purity, thereby affecting both manufacturing costs and the degree of product standardization [42]. Overall, yeast β-glucans provide a sustainable and scalable fermentation-based production model, yet challenges related to manufacturing costs and standardization continue to differentiate them from more commoditized bioactive compounds.

## 7. Applications in the Food Industry

The health benefits of yeast β-glucans along with regulatory approval and the advances in food processing technology have shed light into their incorporation in various conventional products, ranging from supplements to functional foods like beverages, snacks, cereals, etc. Yeast β-glucans have also a water-holding capacity, which allows them to mimic fat and to be used as fat replacers in various foods. Moreover, even if added in small amounts, it can possibly modify a product’s sensory attributes, viscosity, and rheological properties [46].

**Figure 3:**
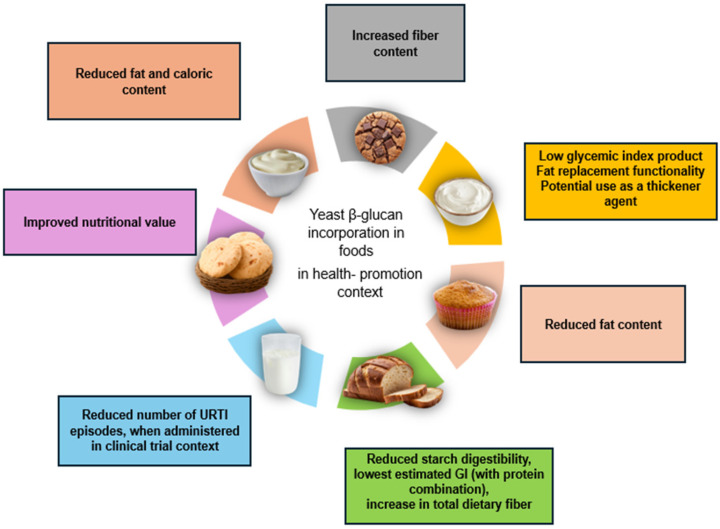
Different food incorporation of yeast beta glucans.

The global yeast β-glucan market is expanding rapidly, driven by growing consumer demand for natural immune-support products and clinically supported ingredients. Several branded yeast β-glucan preparations have established strong positions in the global market [47]. These preparations aim to provide people with an easy way to incorporate the immune-modulating benefits of β-glucans into their nutrition. A wide number of attempts have been made to incorporate yeast β-glucans in foods in terms of designing health-promoting products. Below is an analysis of key research findings on yeast β-glucan application in the food industry [7]. An overview of the incorporation of yeast β-glucans into various food matrices is presented in Figure 3.

### 7.1. Bread and Bakery Products

The studies exploring β-glucan as a functional ingredient have focused on its use in bread and bakery products. A recently published study examined the incorporation of oat, barley, yeast β-glucans (2.5% *w*/*w*), alone and in combination, with protein in whole wheat bread. B-glucan, especially when combined with protein, helps stabilize and strengthen the gluten network in whole wheat dough. This synergy supports bread’s physical structure. While β-glucan can negatively affect some baking qualities, it markedly reduces starch digestibility. Among all formulations tested in the study (sample with protein only, sample with oat β-glucan, sample with oat β-glucan and protein, sample with barley β-glucan, sample with barley β-glucan and protein, sample with yeast β-glucan, sample with yeast β-glucan, and protein and control sample), bread made with yeast β-glucan plus protein had the best overall baking performance. It also demonstrated higher volume, better color, lower hardness and the lowest estimated glycemic index [48]. In the context of making healthy gluten-free products for people with celiac disease, Perez-Quirce et al. investigated the incorporation of β-(1→3), (1→6) soluble and insoluble yeast and filamentous fungi β-glucans, in gluten-free rice dough. Β-glucans were integrated in 0.5–2% *w*/*w*. The β-glucan addition ameliorated the physical quality of the bread by augmenting their specific volume and decreasing their hardness. The insoluble yeast β-glucan incorporation increased the dough’s resistance to deformation but gave a mildly unpleasant flavor and smell [49]. With the aim of exploring the rheological properties of Chinese steamed bread and dough, Lu-Hua et al. implemented oat and yeast β-glucan in quantities of 1–5% of wheat flour. Both types of β-glucans resulted in enhanced gel stability during processing and improved the starch anti-staling performance of bread. The overall quality as well as the nutritional value of Chinese bread was improved with better results shown with 1–2% addition [50]. When added to wheat dough at quantities of 3–9% *w*/*w*, yeast β-glucans lead to dehydration of the gluten network. During dough mixing, water molecules preferentially interact with β-glucans, instead of gluten structure, resulting in alterations in the gluten network [51]. Additionally, when implemented to dough in quantities of 0.25–2% *w*/*w* wheat flour and stored in 4 °C, the end product had high strength and cohesiveness. Among the fortified breads, the product with 0.75% β-glucan seemed to have the least crumb hardness and chewiness and demonstrated the slowest staling rate [52]. The fortification of dough with 0.8–1.2% of weight of flour yeast β-glucans does not seem to have an impact to the viscoelastic properties and the quality of the producing bread [53]. In another study evaluating the enrichment of white bread with β-glucan (2.02 g/100 g flour) and protein/proteolytic enzyme (60 U proteolytic activity/100 g flour) extracts derived from brewer’s yeast, only the β-glucan extract significantly improved the nutritional profile of bread. The β-glucans resulted in a 39% increase in the total dietary fiber content of the bread, while it retained desirable physical characteristics, highlighting yeast β-glucan as a promising functional ingredient that might enhance health-related properties without compromising product quality [54].

An attempt to reduce the total and saturated fat of biscuits has been made, by incorporating yeast and oat β-glucans by Zbikowska et al. They were added in amounts of 1.2–4% of the product with a simultaneous lipid reduction by 5–15%. Biscuits had a significantly higher density, decreased volume, hardness and height, and darker color compared to the control and the oat-biscuits. The yeast β-glucan addition led to a decline in biscuit quality and influenced changes during storage [55]. Similarly, the impact of adding yeast β-glucan on the sensory qualities and bioactive traits of cookies was examined in the context of encouraging fiber intake. Yeast β-glucan flour was incorporated in quantity of 1%, 2% and 4% of wheat flour and was tested for organoleptic properties and biological activity. It was found that the 2% fortification offered the best sensory attributes with an increased lasting freshness and microbiological safety [56]. Beyond cookies, yeast-derived β-glucan supplementation has been incorporated into muffins. Part of the fat had been removed and substituted with yeast β-glucans in 1–4% of the mass product. The replacement had a 1:4 ratio. After baking and storage, muffin characteristics were evaluated. Fat reduction over 40% as well as a β-glucan content beyond 2% seemed to increase the product’s hardness and to deteriorate its sensory characteristics and quality [57].

### 7.2. Dairy Products

Research on the enrichment of foods with β-glucans derived from fungi is not confined only to bakery products. Their addition to dairy products and their functionality have also been studied. Chirsanova et al., Mejri et al., and Raikos et al. have investigated the physicochemical properties of low-fat and non-fat yoghurt enriched with β-glucans obtained from wine sediment and spent brewer’s yeast, respectively. When *S. cerevisiae* β-glucans were added to skimmed-milk yoghurt in 0.1–0.5% *w*/*w*, they reduced fermentation time and achieved the desirable pH quicker than the control sample, without significantly altering the acidity, viscosity and organoleptic characteristics [58]. During application in non-fat yoghurt as a fat replacer (0.5–2% *w*/*w*), the 1.5% *w*/*w* addition seemed to be the most proper in order to have similar rheological properties to a full-fat yoghurt [59].

In a comparable manner, investigating the possibility of thickener, Raikos et al. enhanced yoghurt with 0.2–0.8% *w*/*w* yeast β-glucan. The addition did not alter significantly yoghurt’s physicochemical parameters while it reduced the fermentation time, reaching more quickly the desirable pH. The sensory traits were assessed, and there were not significant differences between the fortified and control sample [60].

Before these studies, Piotrowska et al. investigated the fortification of a 3% fat yoghurt with 0.15% to 0.9% brewer’s spent yeast-derived β-glucans. It was found that in a content of 0.3 per 100 g (0.7 g in a portion) they appeared to be well-tolerated, with no significant impact on sensory qualities or structural stability throughout storage [61].

Regarding milk, a milky beverage has been enriched with yeast β-glucans. More specifically, 250 mg of yeast β-glucans have been added to an ultra-high temperature dairy-based beverage and was administered to marathon runners for daily consumption. Τhe clinical trial is further discussed below [62]. As far as cheese is concerned, the use of yeast glucans in cheese technology has some limitations, as reported in the literature. Yeast is an unfavorable microbe in cheese products and can often cause defects like swelling and cracking. However, other β-glucan sources such as oat and barley have been incorporated in various types of cheeses with positive outcomes regarding technological performance such as enhanced water retention, yield, textural improvements, and microstructural refinement [46].

### 7.3. Other Foods

In the frame of enhancing industrial food properties and increasing their nutritional value, research on other products has been performed. A recently published article reports the fortification of durum wheat pasta with spent brewer’s yeast in amounts of 1–4% *w*/*w* of pasta dough. As the β-glucan content increased, pasta demonstrated lowered toughness and brightness. A reduction in viscoelastic moduli was also observed, accompanied by higher hardness and greater resistance to deformation [63].

Yeast β-glucans were also incorporated into meat batter in 0.5–1.5% *w*/*w* in order to improve meat product’s quality. This addition improved water-holding ability and resulted in enhanced emulsifying capacity, greater emulsion stability and lower hardness and fragility of the end product. An addition up to 3% did not alter the sensory characteristics of meat batter [64]. Another recent study investigated the incorporation of yeast β-glucans in films containing pomegranate juice and their potential application in diabetes prevention. Specifically, spent brewers’ yeast was added in three different quantities (0.5, 1, and 1.5 g of dry β-glucans), along with pomegranate juice, glycerin and sodium alginate forming films. The produced film demonstrated low water vapor permeability, optimal moisture content, and microbial stability, with the addition of 1 g of β-glucans, making it suitable for extending the shelf life of packaged products. Thus, the immunoprotected role of β-glucans in combination with the benefits of pomegranate and the chemical properties of the other substances can formulate a product with a potential role/benefit in diabetes care [65].

Other than that, previous trials have been made to incorporate yeast-derived β-glucans to mayonnaise with the aim of fat replacers. Worrasinchai et al. [66] and Marinescu et al. [67] investigated the replacement of mayonnaise fat with β-glucan isolated from *S. cerevisiae* cells in percentages of 25, 50 and 75%. In both studies, the enriched mayonnaise demonstrated higher storage stability than the full-fat mayonnaise and deteriorated the sensory attributes such as the color and the appearance of the end product. Reduced caloric content, increased water content and stabilizing emulsion properties were achieved. However, sensory evaluation results indicated that mayonnaise formulations in which up to 50% of the fat was substituted with β-glucan were acceptable in terms of texture, taste, odor and overall quality. Table 1 summarizes the main physicochemical, sensorial and nutritional outcomes associated with the enrichment of yeast β-glucans in different foods.

## 8. Analytical Methods for Yeast β-Glucans Assessment and Quantification

Prior to being analyzed, yeast β-glucan must be extracted from *S. cerevisiae’s* or another microorganism’s cell. This includes the isolation of the water-soluble part of the cell after cell disruption. The most common extraction methods of yeast β-glucans are the alkaline extraction, acid extraction, acid–base combined extraction, enzymatic extraction, and enzymatic–alkaline extraction [68]. The optimal extraction method varies according to the structure and the origin of β-glucan [11].

There are some challenges in measuring glycosidic bonds in foods. Quantification of β-glucans is complicated as they may interfere with other glucose-based molecules that are present in many cereal-derived products [69]. When it also comes to measuring (1→3), (1→6) bands, detection must be sensitive enough as yeast β-glucans are frequently present in small amounts in foods [70].

The most commonly used method for determining yeast glucans in foods is the enzymatic colorimetric method. The method was first used in early 1990s by McCleary & Codd to determine mixed-linkage (1→3), (1→4)-β-glucans in cereal grains [71]. It was subsequently adopted as an AOAC Official Method 995.16 for oats and barley [72]. Τhe cereal-based assay was later modified to include additional enzymatic steps (amyloglucosidase and invertase) to hydrolyze α-glucans prior to β-glucan determination of yeast and mushrooms [73]. In this adapted version, β-glucans are solubilized under acidic conditions and extensively hydrolyzed with specific β-glucanases to release glucose units. Total glucose is then quantified colorimetrically via the glucose oxidase–peroxidase (GOPOD) reagent producing a colored compound that is measured spectrophotometrically to give the glucan content on a dry weight basis [74]. This assay kit is commercially available and designed for yeast and mushroom β-glucans incorporated into food products. Its application additionally includes other foods and food matrix [53,60,71].

Despite its utility, the method might occasionally show poor analytical sensitivity and consistency. Heavy presence of high starch in the sample can distort the results. If the content of β-glucans is low, it appears to show negative values at times [70]. It should also be taken into account the fact that acid hydrolysis methods can release glucose from unrelated compounds, potentially interfering with the measurement [69].

Other analytical methods applied to yeast β-glucans include Fourier Transform Infrared Spectroscopy (FT-IR) and Nuclear Magnetic Resonance (NMR) Spectroscopy. FT-IR is mostly applied to verify the functional groups typical of β-glucans. Nevertheless, this technique is predominantly used on isolated yeast β-glucans rather than directly within food matrices [75]. Similarly, NMR has been used in analyzing yeast β-glucan samples. This method confirms structural characterization of β-glucans derived from *S. cerevisiae* and provides detailed insights into the β-(1→3) backbone and β-(1→4) branching points [76]. However, this method, along with FT-IR spectroscopy, exhibits reduced ability in detecting quantitative variation across samples [47].

**Figure 4:**
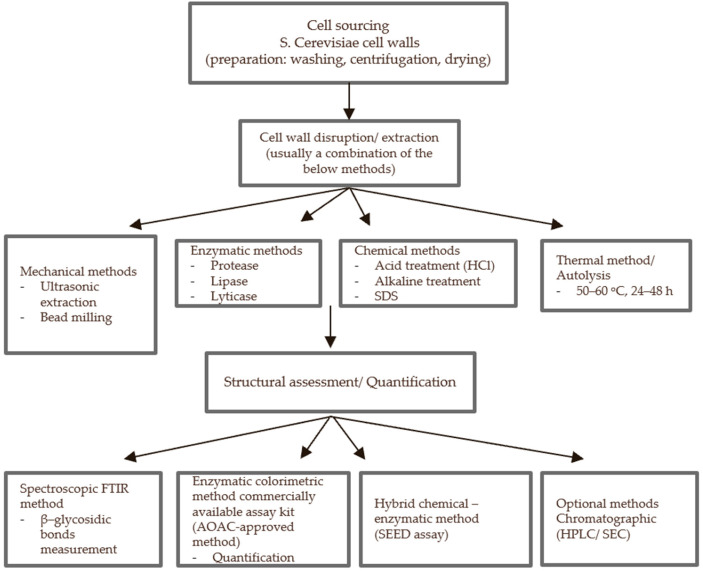
Flowchart pathway from the extraction to the quantification of yeast β-glucan assessment methods. SDS: sodium dodecyl sulfate; FT-IR: Fourier Transform Infrared Spectroscopy; AOAC: Association of Official Analytical Chemists; SEED: Sodium Hypochlorite Extracting and Enzymatic Digesting assay; HPLC/SEC: High Performance Liquid Chromatography/Size Exclusion Chromatography.

Alternative methods have been explored for (1→3), (1→6) glucan content determination. Fluorescent detection with aniline blue was used in bread enriched with yeast β-glucan preparation (0.2–0.5 g/100 g). Despite the overestimation of one preparation, this method showed successful detection of β-(1→3), (1→6) glucans yielding average recoveries of 90%, 96%, 99%, and 105%. Importantly, the presence of cereal-derived β-glucan had no impact on the assay performance [70]. Masahiro et al. developed a novel method of β-glucan quantification, the Sodium Hypochlorite Extracting and Enzymatic Digesting (SEED) assay. This hybrid approach combines chemical extraction and enzymatic quantification for the determination of both (1→3), (1→6) and (1→3), (1→4) β-glucan linkages. The assay was applied in processed foods and polymeric saccharides such as sweet potato, dextrin and laminarin with recovery rate above 90% showing strong accuracy and good reproducibility overall [77]. Figure 4 illustrates the workflow for the extraction and quantification of yeast β-glucans from *S. cerevisiae* cell walls.

## 9. In Vitro Studies

The digestion characteristics of yeast β-glucans have been investigated in vitro. It has been found that when digested, yeast β-glucans are not broken down by saliva, gastric or pancreatic enzymes, and therefore they pass into the colon intact. However, they can be hydrolyzed through fermentation in the gut. It has been also observed that yeast β-glucans have a probiotic action similar to inulin as they support the proliferation of beneficial gut bacteria and restricted dysbiotic shifts, maintaining a balanced gut microbiome [78]. The probiotic function of yeast β-glucans is highlighted in another study which investigates the in vitro digestion of spent brewer’s yeast. Brewer’s yeast enhanced the availability of almost all essential amino acids in comparison to the control sample, and protein biodigestibility was far higher. In addition, the in vitro colon model showed that brewer’s yeast modulated the gut microbiome, leading to higher a-diversity indices, while leaving the β-glucan-rich cell wall fraction largely intact, thereby preserving its capacity to undergo selective microbial fermentation in the colon [79].

In vitro simulated gastrointestinal digestion models serve as vital frameworks for evaluating the digestion behavior, bio-accessibility, and functional interactions of dietary bioactives within complex food matrices. The emulation of the gastrointestinal system processes helps clarify how food structure affects physiological responses [80,81]. Yeast β-glucan has been added into potato starch mixing in 1–3% *w*/*w* of high and low molecular weight, and in vitro digestive properties were studied. This addition resulted in the reduction in the digestibility of potato starch. It also enhanced the thermal stability of starch, decreased its gelatinization viscosity and retarded starch gelatinization. Notably, supplementation with low-molecular-weight β-glucans overall improved the quality of potato starch gel and showed greater enhancement concerning the above effect in comparison to the high-molecular-weight β-glucans [82].

Similarly, pea starch was enriched with filamentous fungi, yeast and oat β-glucans, and the digestive properties were examined. Because of their structure, fungi and yeast β-glucan could form a stronger stability of the mixture in comparison with oat β-glucans. The in vitro digestion indicated an inhibition of starch hydrolysis, which was greater in the fungi β-glucan starch, following the yeast β-glucan and lastly the oat β-glucan starch. The findings suggest that the incorporation of β-glucan into pea starch may contribute to the modulation of postprandial glycemic responses in individuals with diabetes [83].The characteristics of the above studies are summarized in Table 2.

Although research in this area remains modest, existing in vitro digestion studies demonstrate that incorporating yeast β-glucans into food models significantly influences physicochemical properties, enzymatic hydrolysis rates, and potential nutrient or bioactive release.

## 10. In Vivo Studies

Disappointingly, this area using foods fortified with yeast β-glucans has not been extensively studied. Most validated human clinical trials include oral supplementation of yeast β-glucans in the form of preparations, capsules, syrups or powders, with health-promoting characteristics, mainly through the enhancement of the immune system [33].

The clinical trials with food matrices implemented with yeast β-glucans and the investigation of their health impact is limited. The literature reports though two subsequent studies using a beverage enriched with β-glucans and their impact on post-marathon-induced upper respiratory tract infections (URTI).

In the first study, 69 runners consumed 250 mg of the dispersed yeast β-glucan beverage, and 133 runners consumed a calorie- and nutrient-matched placebo beverage for 91 days (45 days before marathon, day of marathon and 45 days post-marathon). Participants who consumed the β-glucan beverage reported significantly fewer days of URTI symptoms (3.43 ± 6.44 days, maximum 27 days) compared to those who consumed the control beverage. In addition, total URTI symptom severity was significantly lower in the β-glucan group (4.52 ± 1.61) than in the control group [62].

The second study extended the results of the first by examining the incorporation of soluble yeast β-glucans in the milky beverage and its effects on URTI in marathon runners. Sixty-nine marathon runners received insoluble β-glucans, 76 received soluble β-glucans in a beverage, and 133 had the placebo beverage. According to the results of the study, the total severity of URTI was significantly reduced in the group receiving insoluble yeast β-glucan compared to the placebo group, whereas no significant difference was observed between the soluble yeast β-glucan group and the placebo. More precisely, severity ratings for nasal discharge were significantly lower in both the insoluble and soluble yeast β-glucan groups contrary to the placebo group. However, sore throat severity was significantly reduced only in the insoluble β-glucan group. The study concludes that both soluble and dispersible yeast β-glucans had differing impacts on exercise-induced URTI in marathon runners when delivered in the food matrix [84].

In the context of examining the influence of yeast β-glucans in young children during flu season, a randomized double-blinded, placebo-controlled trial was designed in China. The study included 156 infants aged 1–4 years-old with at least two URTI episodes in the previous 3 months. Children were divided into three groups receiving daily 75 mg or 35 mg dispersed in water baker’s yeast β-glucans along with sugar and maltodextrin or a placebo beverage containing sugar and maltodextrin for 12 weeks. The β-glucan supplementation significantly reduced both the incidence and duration of infectious and upper-respiratory illnesses in children relative to the placebo. While 85% of placebo group had at least one infection, only 47% and 32% of those taking yeast β-glucan did. Both tested doses (35 mg/day and 75 mg/day) were equally effective, safe, and well tolerated, indicating that 35 mg/day is sufficient for children aged 12–48 months [85].

A smaller clinical trial investigated the relation between yeast β-glucan consumption and stress factors. In this randomized crossover clinical trial, 14 subjects consumed a black koji vinegar (moromisu) containing 300 mg yeast β-glucans for 10 days. Three Profile of Mood State (POMS) surveys were conducted during the trial. The main outcomes of this study were notable decreases in fatigue and confusion, along with strong trends toward reduced anger and increased vigor on the POMS scale. Despite the small sample size and the short supplementation time, significant and near-significant changes in several POMS scale were noted [86]. Table 3 outlines the main features of the aforementioned studies.

## 11. Yeast β-Glucans as “Immunobiotics”: Mechanistic Understanding and Perspectives

The human gastrointestinal tract is colonized by a highly diverse and continually evolving microbial ecosystem that profoundly shapes host physiological processes in both healthy states and disease contexts [18]. Emerging evidence supports that yeast β-glucans exert probiotic-like effects by altering gut microbiota and resulting in gut homeostasis. As has been already mentioned, unlike conventional fiber, yeast β-glucans reach their final colon destination almost intact [78]. The produced SCFAs in gut function as substrates for intestinal colon cells and lower gut’s pH, proliferating beneficial bacteria, such as *Bifidobacterium* sp. and *Lactobacillus* sp., and lowering simultaneously the presence of *Firmicutes* sp. [87]. Studies conducted in animal models with yeast β-glucan supplementation have also shown increases in *Lactobacillus* sp. and *Enterobacteria* sp. with a decrease in Toll-like receptors on intestinal cells and lowered transcription of inflammatory mediators [88]. Furthermore, administration of yeast β-glucan in mice with obesity and type 2 diabetes counteracted some of the metabolic damage caused by high-fat nutrition. Beyond proliferating beneficial gut bacteria populations, the supplementation resulted in decreasing mitochondrial and proteosynthesis impairment and oxidative stress factors [89].

The reshapes in microbial ecology attributed to yeast β-glucans are related to their anti-inflammatory pathways and thus the immune-modulatory effects. This microbiota-related immunoregulation contributes to “trained immunity”, a memory situation in which innate immune cells are long-term reprogrammed once there is an initial stimulus [90]. Trained β-glucan-mediated activation of the Dectin-1 receptor has been considered the main driver of trained immunity through reprogramming of innate immune cells [91]. The interaction of yeast β-glucans with immune receptors and gut-associated lymphoid tissue (GALT) initiates the production of pro- and anti-inflammatory cytokines. Then β-glucans are ingested and processed by dendritic cells and macrophages which stimulate T helper cytokines (Th1, Th2) and T-cells. B-cells and IgA secretion are simultaneously activated contributing to reinforcing the immune defenses of mucosal tissues and the integrity of the epithelial barrier [92]. Collectively, these processes illustrate how yeast-derived β-glucans directly influence immune responses by engaging cellular receptors and supporting antigen presentation in the intestinal environment.

Beyond the above direct interconnections of yeast β-glucans with immune cell receptors, SCFAs serve as an important indirect immunomodulatory pathway that should not be neglected. Recent evidence underlines the role of the microbiome as an additional layer of immunoregulation in which microbiota-derived metabolites reshape monocyte and macrophage function by modulating chromatin accessibility and cellular metabolism [93]. SCFAs and particularly butyric acid and to a smaller degree propionic acid are capable of inhibiting histone deacetylase (HDAC) in epithelial cells promoting epigenetic modifications that enhance regulatory T-cell differentiation and anti-inflammatory cytokine production. Thus, the expression of genes linked to immune tolerance and anti-inflammatory responses is enhanced [87]. In addition to shaping gene expression through epigenetic mechanisms, short-chain fatty acids also regulate immune function by binding to G protein-coupled receptors. These include GPR41, GPR43, and GPR109 and are located on the epithelial and various immune cells. Activation of these signaling pathways influences inflammasome activation, alters cytokine production, and controls the migration of neutrophils to sites of inflammation [94]. Therefore, the microbiota-mediated production of SCFAs represents a crucial intermediary mechanism through which yeast β-glucans extend their immunomodulatory capacity and barrier-protective effects.

In total, these findings support a dual model in which yeast β-glucans act as multifunctional “immunobiotics”. The indirect, SCFA-driven pathway is not merely complementary but fundamentally synergistic with direct receptor-mediated signaling, collectively defining the full immunological spectrum of yeast β-glucan activity. However, future investigations should focus on clarifying the respective contributions of all immune-microbial mechanisms and pathways in human clinical contexts. In addition, further studies are needed to elucidate how structural characteristics of β-glucans—particularly branching degree and molecular weight—influence microbiota-associated immunomodulatory responses.

## 12. Conclusions

Yeast β-glucans derived from *S. cerevisiae* hold promises as a bioactive polysaccharide for implementing various foods with proven beneficial effects for humans. Their distinctive β-(1→3), (1→6)-linked structure underlies a dual immunomodulation through direct and indirect gut-mediated pathway. Their addition in various foods has already shown beneficial effects on product quality and stability, and in vitro and preliminary in vivo studies suggest immunomodulatory properties. Nevertheless, available evidence is still limited and fragmented, with most human data derived from supplement-based interventions rather than food-enriched products. Establishing standardized analysis methods, together with well-designed clinical trials with food matrices, is crucial to determine whether the beneficial effects demonstrated by purified yeast β-glucans are maintained in complex food systems. With continued scientific validation and technological refinement, they hold substantial potential for the development of next-generation functional foods targeting immune resilience, metabolic health, and gut homeostasis.

## Figures and Tables

**Table 1:** Physicochemical, sensorial and nutritional outcomes of yeast β-glucan incorporation into various food products.

Food Category	Food Product	B-glucan Type and Dosage	Technological/Physicochemical Effects	Organoleptic Effects	Nutritional/Functional Outcomes	Reference
Bread and Bakery	Whole wheat bread (alone or in combination with protein)	2.5% *w*/*w* oat, barley and yeast β-glucans alone and in combination with protein	Strengthened gluten network and improved structure (with the combination of protein and β-glucan), higher loaf volume and reduced hardness with yeast β-glucans	Better color, improved overall baking performance especially with yeast β-glucans	Reduced starch digestibility, lowest estimated GI (with protein combination)	[48]
	Gluten-free rice bread	0.5–2% *w*/*w* insoluble yeast and filamentous fungi β-glucans	Increased specific volume, decreased hardness, increased dough resistance with yeast β-glucan addition	Insoluble yeast β-glucans caused mildly unpleasant odor and flavor	Improved physical quality for gluten-free products	[49]
	Chinese steamed bread	Oat and yeast β-glucan in 1–5% of wheat flour	Enhanced dough elasticity, increased specific volume, reduced product rigidity and attenuated starch retrogation in both β-glucan forms (1–2% addition)	Improved overall quality (optimal at 1–2%)	Improved nutritional value	[50]
	Wheat dough during the mixing	3–9% *w*/*w* barley and beer yeast	Dehydration of gluten network, mechanical disruption of the dough structure in both β-glucan forms	Not reported	Not reported	[51]
	Wheat bread (stored at 4 °C)	0.25–2% *w*/*w* yeast β-glucan	Increased strength and cohesiveness, slowest product staling (optimal at 0.75%)	Reduced crumb hardness and chewiness (optimal 0.75%)	Delayed staling	[52]
	Wheat bread	Yeast β-glucan at 0.8–1.2% of flour weight	No significant impact on viscoelastic properties	No quality deterioration	Potential to be used as a functional ingredient	[53]
	White wheat bread	Yeast β-glucan extract 2.02 g/100 g flour and protein/proteolytic enzymes	Maintained desirable physical characteristics	Better color with β-glucan addition	39% increase in total dietary fiber, health-promoting potential	[54]
	Biscuits (fat reduced)	1.3–4% *w*/*w* yeast or oat β-glucans	Increased density, decreased volume, height and hardness	Darker color of yeast β-glucan biscuits, decline in quality during storage at higher levels of addition	Reduced total and saturated fat	[55]
	Cookies	Yeast β-glucans at 1–4% of wheat flour	Improved freshness retention, microbiological safety	Best sensory attributes at 2% addition	Health-promoting potential due to fiber intake	[56]
	Muffins	1–4% *w*/*w* yeast β-glucans	Increased hardness when >2% or fat reduction >40%	Deteriorated sensory quality during storage and acceptance at higher levels of addition	Fat reduction	[57]
Dairy Products	Skimmed-milk yoghurt	0.1–0.5% *w*/*w* yeast β-glucan	Reduced fermentation time, faster pH reduction, no significant change in viscosity	No significant sensory alteration	Health-promoting and low glycemic index product	[58]
	Non-fat yoghurt	0.5–2% *w*/*w* yeast β-glucan	Increased oil and water binding, emulsion stabilizing capability, similar rheological properties to full-fat yoghurt at 1.5%	Desirable sensory properties up to 1.5% enrichment	Fat replacement functionality	[59]
	Skimmed-milk yoghurt	0.2–0.8% *w*/*w* yeast β-glucan	Increased product hardness, reduced fermentation time, stable physicochemical parameters	No significant difference compared to control	Potential use as a thickener agent	[60]
	3% fat yoghurt	0.15–0.9% *w*/*w* yeast β-glucan	Maintained structural stability during storage (up to 0.3% addition)	No adverse sensory impact up to 0.3% addition	Health-promoting product	[61]
	UHT dairy beverage	250 mg per serving	Not reported	Not reported	Administered in clinical trial context, reduced number of URTI episodes	[62]
Other Foods	Durum wheat pasta	1–4% *w*/*w* yeast β-glucan	Reduced viscoelastic moduli, increased hardness and deformation resistance	Lower pasta yellowness	Nutritional enrichment	[63]
	Meat batter	0.5–1.5% *w*/*w* yeast β-glucan	Improved water holding, enhanced emulsion stability, reduced hardness and fragility	No significant sensory alteration	Improved product quality	[64]
	Edible films (with pomegranate juice)	0.5–1.5 dry yeast β-glucan	Low water vapor permeability, microbial stability, optimal moisture at 1 g β-glucan	Not reported	Potential role in diabetes prevention, shelf-life extension	[65]
	Mayonnaise	25–75% fat replacement	Increased water content, improved emulsion stability, higher storage stability	Sensory deterioration at higher replacement levels, (≤50% acceptable)	Reduced fat and caloric content	[66,67]

**Table 2:** Summary of in vitro fermentation studies using yeast β-glucans.

Study Design/Focus	Food Model System	Yeast Beta Glucan Characteristics	Main Outcome	Reference
Gastrointestinal digestion and fermentability	In vitro digestion and colon fermentation models	Yeast β-glucans	Resistant to salivary, gastric, and pancreatic enzymes; reached the colon intact; fermented by gut microbiota; exhibited prebiotic-like activity like inulin, supporting beneficial bacteria	[78]
In vitro protein digestibility, amino acid inaccessibility, and protein quality of processed brewer’s spent yeast was explored using the static INFOGEST in vitro model	In vitro digestion and colon fermentation model	β-glucan-rich cell wall fraction.	Increased essential amino acid availability and protein biodigestibility; enhanced gut microbial α-diversity; preserved β-glucan structure for selective fermentation	[79]
Effect on potato starch digestibility	Potato starch model	High- and low-molecular-weight yeast β-glucans (1–3% *w*/*w*)	Reduced starch digestibility; improved thermal stability; decreased gelatinization viscosity; delayed gelatinization; low-MW β-glucans showed superior effects	[82]
Pasting, gelation, and digestive properties of pea starch	Pea-starch-enriched with filamentous fungi, yeast and oat β-glucans	Yeast β-glucans (0.25–1% *w*/*w*)	Reduced starch digestibility; improved thermal stability; decreased gelatinization viscosity; delayed gelatinization; low-MW β-glucans showed superior impact	[83]

**Table 3:** Summary of in vivo clinical trials using yeast β-glucans incorporated in beverages.

STUDY Type	Population	Food Matrix	B-Glucan Type and Dosage	Supplementation Time	Main Outcome	Reference
Randomized double-blind, placebo-control study	Marathon runners (*n* = 202)	Dairy-based beverage	250 mg/day dispersible yeast β-glucans	91 days	Significantly fewer days with URTI symptoms and reduced total symptom severity compared to placebo	[62]
Randomized double-blind, placebo-control study	Marathon runners (*n* = 278)	Dairy-based beverage	250 mg/day soluble or insoluble yeast β-glucans	91 days	Reduced total URTI severity in insoluble β-glucan group; both forms reduced nasal discharge severity; insoluble form reduced sore throat severity	[84]
Randomized, double-blind, placebo-controlled	Children aged 1–4 years (*n* = 174)	Beverage (β-glucans dispersed in water)	35 or 75 mg/day baker’s yeast β-glucans	12 weeks	Reduced incidence and duration of common childhood infectious illness episodes; both doses equally effective and well tolerated	[85]
Randomized crossover trial	Healthy adults (*n* = 14)	Black koji vinegar (moromisu) beverage	300 mg/day soluble baker’s yeast β-glucans	10 days with an 11-day washout	Significant reductions in fatigue and confusion; trends toward reduced anger and increased vigor	[86]

## Data Availability

The original contributions presented in this study are included in the article. Further inquiries can be directed to the corresponding author.

## References

[1] Granato D., Barba F.J., Lorenzo J.M., Cruz A.G., Putnik P. (2020). Functional Foods: Product Development, Technological Trends, Efficacy Testing, and Safety. Annu. Rev. Food Sci. Technol..

[2] (2024). Functional Foods (2024–2030)—Functional Foods Market Size, Share & Trends Analysis Report by Ingredient (Carotenoids, Prebiotics & Probiotics, Fatty Acids, Dietary Fibers), by Product, by Application, by Region, and Segment Forecasts. Grand View Research. https://www.grandviewresearch.com/industry-analysis/functional-food-market.

[3] Han B., Baruah K., Cox E., Vanrompay D., Bossier P. (2020). Structure-Functional Activity Relationship of β-Glucans From the Perspective of Immunomodulation: A Mini-Review. Front. Immunol..

[4] Zhong X., Wang G., Li F., Fang S., Zhou S., Ishiwata A., Tonevitsky A.G., Shkurnikov M., Cai H., Ding F. (2023). Immunomodulatory Effect and Biological Significance of β-Glucans. Pharmaceutics.

[5] Luo J., Chen D., Mao X., He J., Yu B., Cheng L., Zeng D. (2019). Purified β-glucans of Different Molecular Weights Enhance Growth Performance of LPS-challenged Piglets via Improved Gut Barrier Function and Microbiota. Animals.

[6] Johnston C.J.H., Ledwith A.E., Lundahl M.L., Charles-Messance H., Hackett E.E., O’sHaughnessy S.D., Clegg J., Prendeville H., McGrath J.P., Walsh A.M. (2024). Recognition of yeast b-glucan particles triggers immunometabolic signaling required for trained immunity. iScience.

[7] Sarkar N., Mahajan A.A., Pathak S., Seth P., Chowdhury A., Ghose I., Das S., Chowdhury R., Bera A., Dey A. (2025). Beta-Glucans in Biotechnology: A Holistic Review with a Special Focus on Yeast. Bioengineering.

[8] Thomas S., Rezoagli E., Abidin I.Z., Major I., Murray P., Murphy E.J. (2022). β-Glucans from Yeast—Immunomodulators from Novel Waste Resources. Appl. Sci..

[9] Du B., Meenu M., Liu H., Xu B. (2019). A Concise Review on the Molecular Structure and Function Relationship of β-Glucan. Int. J. Mol. Sci..

[10] Zhu F., Du B., Bian Z., Xu B. (2015). Beta-Glucans from edible and medicinal mushrooms: Characteristics, physicochemical and biological activities. J. Food Compos. Anal..

[11] Chioru A., Chirsanova A. (2023). β-Glucans: Characterization, Extraction Methods, and Valorization. Food Nutr. Sci..

[12] EFSA Panel on Dietetic Products, Nutrition and Allergies (NDA) (2011). Scientific Opinion on the safety of ‘yeast beta-glucans’ as a Novel Food. EFSA J..

[13] Yuan H., Lan P., He Y., Li C., Ma X. (2020). Effect of the Modifications on the Physicochemical and Biological Properties of β-Glucan—A Critical Review. Molecules.

[14] Xu Z., Meng X., Bin Y., Li H., Qin Y., Qi Z. (2024). Exploring the therapeutic potential of yeast β-glucan: Prebiotic, anti-infective, and anticancer properties—A review. Int. J. Biol. Macromol..

[15] Fu W., Zhao G., Liu J. (2022). Effect of preparation methods on physiochemical and functional properties of yeast β-glucan. Food Sci. Technol. Int..

[16] Kaur R., Sharma M., Ji D., Xu M., Agyei D. (2020). Structural Features, Modification, and Functionalities of Beta-Glucan. Fibers.

[17] Avramia I., Amariei S. (2021). Spent Brewer’s Yeast as a Source of Insoluble β-Glucans. Int. J. Mol. Sci..

[18] Singh R.P., Bhardwaj A. (2023). β-glucans: A potential source for maintaining gut microbiota and the immune system. Front. Nutr..

[19] Guo W., Gu X., Tong Y., Wang X., Wu J., Chang C. (2019). Protective effects of mannan/β-glucans from yeast cell wall on the deoxyniyalenol-induced oxidative stress and autophagy in IPEC-J2 cells. Int. J. Biol. Macromol..

[20] Yu C., Chen H., Du D., Lv W., Li S., Li D., Xu Z., Gao M., Hu H., Liu D. (2021). β-Glucan from *Saccharomyces cerevisiae* alleviates oxidative stress in LPS-stimulated RAW264.7 cells via Dectin-1/Nrf2/HO-1 signaling pathway. Cell Stress Chaperones.

[21] Du B., Lin C., Bian Z., Xu B. (2015). An insight into anti-inflammatory effects of fungal beta-glucans. Trends Food Sci. Technol..

[22] Bacha U., Nasir M., Iqbal S., Anjum A.A. (2017). Nutraceutical, Anti-Inflammatory, and Immune Modulatory Effects of β-Glucan Isolated from Yeast. BioMed Res. Int..

[23] Han F., Fan H., Yao M., Yang S., Han J. (2017). Oral administration of yeast b -glucan ameliorates inflammation and intestinal barrier in dextran sodium sulfate-induced acute colitis. J. Funct. Foods.

[24] Cheng J., Zhang G., Liu L., Luo J., Peng X. (2023). Anti-inflammatory activity of β-glucans from different sources before and after fermentation by fecal bacteria in vitro. J. Sci. Food Agric..

[25] Zhu Z., He L., Bai Y., Xia L., Sun X., Qi C. (2023). Yeast β-glucan modulates macrophages and improves antitumor NK-cell responses in cancer. Clin. Exp. Immunol..

[26] European Commission (2011). Commission Implementing Decision of 24 November 2011. Off. J. Eur. Union.

[27] European Commission (2017). Commission Implmenting Decision (EU) 2017/2078 of 10 November 2017. Off. J. Eur. Union.

[28] U.S. Food and Drug Administration (2008). GRN No. 239 Bakers Yeast Beta-Glucan [Internet]. https://hfpappexternal.fda.gov/scripts/fdcc/index.cfm?set=GRASNotices&id=239&sort=GRN_No&order=DESC&startrow=1&type=basic&search=Glucan.

[29] (2026). World Health Organization-Healthy Diet [Internet]. https://www.who.int/news-room/fact-sheets/detail/healthy-diet.

[30] Government of Canada (2024). Completed Safety Assessments of Novel Foods Including Genetically Modified (GM) Foods Sourvisiae^®^. https://www.canada.ca/en/health-canada/services/food-nutrition/genetically-modified-foods-other-novel-foods/approved-products/sourvisiae.html.

[31] Therapeutic Goods Administration (1990). Therapeutic Goods Regulations 1990, Department of Health, Disability and Ageing. https://www.tga.gov.au/resources/legislation/therapeutic-goods-regulations-1990.

[32] Iruoghene G., Njolke A., Ali A.B.M., Othuke P., Yousif E., Fegor E., Augustina U., Zainulabdeen K., Oghenewogaga J., Efeoghene A.A.E. (2025). A critical review on the impacts of β-glucans on gut microbiota and human health. Microbe J..

[33] Vlassopoulou M., Yannakoulia M., Pletsa V., Zervakis G.I., Kyriacou A. (2021). Effects of fungal beta-glucans on health—A systematic review of randomized controlled trials. Food Funct..

[34] Sasaki H., Masutomi H., Kobayashi Y., Toyohara K. (2025). Evaluation of the Fermentation Characteristics of Containing Granola and Short- Chain Fatty Acid Production in an In Vitro Gut Microbiota Model. Food Sci. Nutr..

[35] Reay S.L., Ferreira A.M., Hilkens C.M.U., Novakovic K. (2025). The Paradoxical Immunomodulatory Effects of Chitosan in Biomedicine. Polymers.

[36] Singh A., Yau Y.F., Leung K.S., El-nezami H., Lee J.C.-Y. (2020). Interaction of Polyphenols as Antioxidant and Anti-Inflammatory Compounds in Brain—Liver—Gut Axis. Antioxidants.

[37] Liu Y., Wu Q., Wu X., Attia S., Gong F., Hu J., Luo W., Zhou M., Pan Y., Yan Y. (2021). Structure, preparation, modification, and bioactivities of β -glucan and mannan from yeast cell wall: A review. Int. J. Biol. Macromol..

[38] Canazza E., Grauso M., Mihaylova D. (2025). Techno-Functional Properties and Applications of Inulin in Food Systems. Gels.

[39] Zhen H., Qian H., Liu X., Tan C. (2024). Fructooligosaccharides for Relieving Functional Constipation: A Systematic Review and Meta-Analysis of Randomized Controlled Trials. Foods.

[40] (2021). Committee on Toxicity of Chemicals in Food, Consumer Products and the Environment Additional Information Requested by the Committee on Allergenicity of Chitin and Chitosan Based BBFCMs. https://www.semanticscholar.org/paper/COMMITTEE-ON-TOXICITY-OF-CHEMICALS-IN-FOOD%2C-AND-THE/d0fb985c43b050a9389a1eb4572d4f76e2c85eab.

[41] Duda-chodak A. (2023). Possible Side Effects of Polyphenols and Their Interactions with Medicines. Molecules.

[42] Morales D. (2023). Food By-Products and Agro-Industrial Wastes as a Source of β -Glucans for the Formulation of Novel Nutraceuticals. Pharmaceuticals.

[43] Tsigoriyna L., Stefanov S., Armenova N., Petrova P. (2024). Microbial Conversion of Inulin to Valuable Products: The Biorefinery Concept. Fermentation.

[44] Grifoll V., Bravo P., Nieves M., Margarita P., Garc M., Larran A., Lizundia E. (2024). Environmental Sustainability and Physicochemical Property Screening of Chitin and Chitin-Glucan from 22 Fungal Species. ASC Sustain. Chem. Eng..

[45] Palos-hern A., Gonz A.M. (2025). Latest Advances in Green Extraction of Polyphenols from Plants, Foods and Food By-Products. Molecules.

[46] Mykhalevych A., Polishchuk G., Nassar K., Osmak T., Buniowska-olejnik M. (2022). β-Glucan as a Techno-Functional Ingredient in Dairy and Milk-Based Products—A Review. Molecules.

[47] Boutros J.A., Magee A.S., Cox D. (2022). Comparison of structural differences between yeast β-glucan sources from different strains of saccharomyces cerevisiae and processed using proprietary manufacturing processes. Food Chem..

[48] Zeng F., Hu Z., Yang Y., Jin Z., Jiao A. (2024). Regulation of baking quality and starch digestibility in whole wheat bread based on β-glucans and protein addition strategy: Significance of protein-starch-water interaction in dough. Int. J. Biol. Macromol..

[49] Perez-quirce S., Caballero P.A., Vela A.J., Villanueva M., Ronda F. (2018). Impact of yeast and fungi (1/3)(1/6)-β-glucan concentrates on viscoelastic behavior and bread making performance of gluten-free rice-based doughs. Food Hydrocoll..

[50] Pan L.-H., Luo S.-Z., Liu F., Luo J.-P. (2018). Comparison of rheological properties of dough and antistaling characteristics of Chinese Steamed Bread containing b-glucan from yeast or oat. Cereal Chem..

[51] Welc-stanowska R., Karp S., Kurek M., Miś A., Nawrocka A. (2023). Effect of β-glucans on water redistribution and gluten structure in a model dough during the mixing process. Int. Agrophysics.

[52] Suwannarong S., Wongsagonsup R., Suphantharika M. (2020). Effect of spent brewer’s yeast β-D-glucan on properties of wheat flour dough and bread during chilled storage. Int. J. Biol. Macromol..

[53] Marukhnenko S., Gerasimov A., Ivanova V., Golovinskaia O., Antontceva E., Pokatova O., Morozov A., Shamtsyan M. (2020). *Saccharomyces cerevisiae* yeasts β-glucan influence on wheat dough rheological properties. E3S Web Conf..

[54] Martins Z.E., Pinho O., Fereeira I.M.P.L.V.O. (2018). Impact of new ingredients obtained from brewer ’ s spent yeast on bread characteristics. J. Food Sci. Technol..

[55] Zbikowska A., Kowalska M., Zbikowska K., Onacik-Gur S., Lempicka U., Turek P. (2022). Study on the Incorporation of Oat and Yeast β-Glucan into Shortbread Biscuits as a Basis for Designing Healthier and High Quality Food Products. Molecules.

[56] Bacha U., Nasir M., Iqbal S., Anjum A.A. (2018). Influence of Yeast β-Glucan on Cookies Sensory Characteristics and Bioactivities. J. Chem..

[57] Kupiec M., Szymanska I., Osytek K., Zbikowska A., Kowalska M., Marciniak-lukasiak K., Rutkowska J. (2020). Microbial β-glucan Incorporated into Muffins: Impact on Quality of the Batter and Baked Products. Agriculture.

[58] Chrisanova A.I., Boistean A.V., Chiselita N., Siminiuc R. (2021). Impact of yeast sediment beta-glucans on the quality indices of yoghurt. Food Syst..

[59] Mejri W., Bornaz S., Sahli A. (2015). Formulation of non-fat yoghurt with β-glucan from spent brewer’s yeast. J. Hyg. Eng. Des..

[60] Raikos V., Grant S.B., Hayes H., Ranawana V. (2018). Use of β-glucan from spent brewer’s yeast as a thickener in skimmed yogurt: Physicochemical, textural, and structural properties related to sensory perception. J. Dairy Sci..

[61] Piotrowska A., Waszkiewicz-robak B., Świderski F. (2009). Possibility of Beta-glucan from spent brewer’s yeast addition to yoghurts. Polish J. Food Nutr. Sci..

[62] Mah E., Kaden V.N., Kelley K.M., Liska D.J. (2020). Beverage Containing Dispersible Yeast β-Glucan Decreases Cold/Flu Symptomatic Days After Intense Exercise: A Randomized Controlled. J. Diet. Suppl..

[63] Ungureanu-Iuga M., Avramia I. (2024). Pasta fortified with β-glucan Isolated from brewer’s yeast (*Saccharomyces cerevisiae*) by-product. J. Cereal Sci..

[64] Apostu P.M., Elena T., Anca M., Nicolau I. (2017). Technological and sensorial role of yeast b-glucan in meat batter reformulations. J. Food Sci. Technol..

[65] Avramia I., Amariei S. (2022). Formulation, Characterization and Optimization of β-Glucan and Pomegranate Juice Based Films for Its Potential in Diabetes. Nutrients.

[66] Worrasinchai S., Suphantharika M., Pinjai S., Jamnong P. (2006). β-Glucan prepared from spent brewer’s yeast as a fat replacer in mayonnaise. Food Hydrocoll..

[67] Marinescu G., Stoicescu A., Patrascu L. (2011). The preparation of mayonnaise containing spent brewer’s yeast β-glucan as a fat replacer. Rom. Biotechnol. Lett..

[68] Yang W., Huang G. (2021). Extraction methods and activities of natural glucans. Trends Food Sci. Technol..

[69] Apostu P.M., Nicolau A.I., Mihociu T.E. (2017). Enzymatic Quantification of β -glucan in a Finely Comminuted Meat Product System. Food Anal. Methods.

[70] Rieder A., Ballance S., Böcker U., Knutsen S. (2018). Quantification of 1,3-β-D-glucan from yeast added as a functional ingredient to bread. Carbohydr. Polym..

[71] McCleary B.V., Codd R. (1991). Measurement of (1→3),(1→4)-β-D-glucan in barley and oats: A streamlined enzymic procedure. J. Sci. Food Agric..

[72] McCleary B.V., MugForg D.V. (1997). Determination of β-Glucan in Barley and Oats by Streamlined Enzymatic Method: Summary of Collaborative Study. J. AOAC Int..

[73] McCleary B.V., Draga A. (2016). Measurement of β-Glucan in Mushrooms and Mycelial Products. J. AOAC Int..

[74] Danielson M.E., Dauth R., Elmasry N.A., Magee R.R.L., Magee A.S., Will P.M. (2010). Enzymatic Method To Measure β-(1, 3)-β-(1, 6)-Glucan Content in Extracts and Formulated Products (GEM Assay). J. Agric. Food Chem..

[75] Bikmurzin R., Bandzeviciute R., Marsalka A., Maneikis A. (2022). FT-IR Method Limitations for β-Glucan Analysis. Molecules.

[76] Yan M., Wang X., Zhang J., Yang S., Pan C., Liu S. (2025). Extraction, structural characterization, and in vitro immunomodulatory activities of *Saccharomyces cerevisiae* spore wall β-glucan. J. Agric. Food Res..

[77] Ide M., Okumura M., Koizumi K., Kumagai M., Yoshida I. (2018). Novel Method to Quantify β-Glucan in Processed Foods: Sodium Hypochlorite Extracting and Enzymatic Digesting (SEED) Assay. J. Agric. Food Chem..

[78] Wang H., Chen G., Li X., Zheng F., Zeng X. (2020). Yeast β-glucan, a potential prebiotic, showed a similar probiotic activity to inulin. Food Funct..

[79] Jaeger A., Nyhan L., Sahin A.W., Zannini E., Meehan D., Li J., O’tOole P.W., Arendt E.K. (2025). In vitro digestibility of bioprocessed brewer’s spent yeast: Demonstrating protein quality and gut microbiome modulation potential. Food Res. Int..

[80] Zhou H., Tan Y., McClements D.J. (2023). Applications of the INFOGEST In Vitro Digestion Model to Foods: A Review. Annu. Rev. Food Sci. Technol..

[81] Brodkorb A., Egger L., Alminger M., Alvito P., Assunção R., Ballance S., Bohn T., Bourlieu-Lacanal C., Boutrou R., Carrière F. (2019). INFOGEST static in vitro simulation of gastrointestinal food digestion. Nat. Protoc..

[82] Zhang L., Dong L., Zhang H., Zhang Y., Xia M. (2024). Effects of yeast β-glucan on gelatinization, structure and digestibility of potato starch. J. Sci. Food Agric..

[83] Cui Y., Han X., Huang X., Xie W., Zhang X., Zhang Z., Yu Q., Tao L., Li T., Li S. (2023). Effects of different sources of β-glucan on pasting, gelation, and digestive properties of pea starch. Food Hydrocoll..

[84] Trial RPcontrolled Mah E., Kaden V.N., Kelley K.M., Liska D.J. (2019). Soluble and Insoluble Yeast b-Glucan Differentially Affect Upper Respiratory Tract Infection in Marathon Runners: A Double-Blind, Randomized Placebo-Controlled Trial. J. Med. Food.

[85] Meng F. (2016). Baker’s Yeast Beta-Glucan Decreases Episodes of Common Childhood Illness in 1 to 4 Year Old Children during Cold Season in China. J. Nutr. Food Sci..

[86] Ojiri Y., Endoh H., Okumoto T., Atsuta K. (2015). Randomized, double-blind, placebo-controlled, crossover study to evaluate the effects of beta-1, 3/1, 6 glucan on stress associated with daily lifestyle in healthy subjects. Funct. Foods Heal Dis..

[87] Edo G.I., Mafe A.N., Ali A.B.M., Akpoghelie P.O., Yousif E., Isoje E.F., Igbuku U.A., Zainulabdeen K., Owheruo J.O., Essaghah A.E.A. (2025). Mechanistic insights into β-glucans and gut microbiota interactions for enhancing human health. Discov. Food.

[88] Wenrui Z., Liu Y., Shao Y., Ma Y., Wu Y., Guo F., Abbas W., Guo Y., Wang Z. (2021). Yeast β-Glucan Altered Intestinal Microbiome and Metabolome in Older Hens. Front. Microbiol..

[89] Mitchelson K.A.J., Tran T.T.T., Dillon E.T., Vlckova K., Harrison S.M., Ntemiri A., Cunningham K., Gibson I., Finucane F.M., O′COnnor E.M. (2022). Yeast β-Glucan Improves Insulin Sensitivity and Hepatic Lipid Metabolism in Mice Humanized with Obese Type 2 Diabetic Gut Microbiota. Mol. Nutr. Food Res..

[90] Ajit J., Chen Q., Ung T., Rosenberger M., Kim J., Solanki A., Shen J., Kahn A.P.E. (2025). β-glucan induced trained immunity enhances antibody levels in a vaccination model in mice. PLoS ONE.

[91] Renke G., Baesso T., Paes R., Renke A. (2022). β-Glucan “Trained Immunity“ Immunomodulatory Properties Potentiate Tissue Wound Management and Accelerate Fitness Recover. ImmunoTargets Ther..

[92] Castro E.D.M., Calder P.C., Roche H.M. (2021). β-1, 3/1, 6-Glucans and Immunity: State of the Art and Future Directions. Mol. Nutr. Food Res..

[93] Ferenc K., Sokal-dembowska A., Helma K., Jarmakiewicz-czaja S. (2024). Modulation of the Gut Microbiota by Nutrition and Its Relationship to Epigenetics. Int. J. Mol. Sci..

[94] Koh A., De Vadder F., Kovatcheva-Datchary P., Bäckhed F. (2016). Review From Dietary Fiber to Host Physiology: Short-Chain Fatty Acids as Key Bacterial Metabolites. Cell.

